# Nitric oxide synthase expression in *Pseudomonas koreensis* MME3 improves plant growth promotion traits

**DOI:** 10.1007/s00253-024-13029-1

**Published:** 2024-02-15

**Authors:** María M. Labarthe, Guillermo A. Maroniche, Lorenzo Lamattina, Cecilia M. Creus

**Affiliations:** 1 Facultad de Ciencias Agrarias, Universidad Nacional de Mar del Plata, Balcarce, Buenos Aires Argentina; 2 Consejo Nacional de Investigaciones Científicas y Técnicas (CONICET), Ciudad Autónoma de Buenos Aires, Argentina; 3 IIB, Universidad Nacional de Mar del Plata, Mar del Plata, Buenos Aires, Argentina

**Keywords:** *Pseudomonas*, Nitric oxide synthase, Nitrogen, Plant growth–promoting rhizobacteria, *Brachypodium*

## Abstract

**Abstract:**

The development of novel biotechnologies that promote a better use of N to optimize crop yield is a central goal for sustainable agriculture. Phytostimulation, biofertilization, and bioprotection through the use of bio-inputs are promising technologies for this purpose. In this study, the plant growth–promoting rhizobacteria *Pseudomonas koreensis* MME3 was genetically modified to express a nitric oxide synthase of *Synechococcus* SyNOS, an atypical enzyme with a globin domain that converts nitric oxide to nitrate. A cassette for constitutive expression of *synos* was introduced as a single insertion into the genome of *P. koreensis* MME3 using a miniTn*7* system. The resulting recombinant strain MME3:SyNOS showed improved growth, motility, and biofilm formation. The impact of MME3:SyNOS inoculation on *Brachypodium distachyon* growth and N uptake and use efficiencies under different N availability situations was analyzed, in comparison to the control strain MME3:c. After 35 days of inoculation, plants treated with MME3:SyNOS had a higher root dry weight, both under semi-hydroponic and greenhouse conditions. At harvest, both MME3:SyNOS and MME3:c increased N uptake and use efficiency of plants grown under low N soil. Our results indicate that *synos* expression is a valid strategy to boost the phytostimulatory capacity of plant-associated bacteria and improve the adaptability of plants to N deficiency.

**Key points:**

*• synos expression improves P. koreensis MME3 traits important for rhizospheric colonization*

*• B. distachyon inoculated with MME3:SyNOS shows improved root growth*

*• MME3 inoculation improves plant N uptake and use efficiencies in N-deficient soil*

## Introduction

In the last decade, worldwide population growth increased the demand for food, mainly grain crops such as wheat, which in turn fostered the use of fertilizers to maximize the yields of agricultural production (Man et al. 2021). Nitrogen (N) is a main limiting factor in crop productivity and the principal macronutrient supplemented to crops (Garnett et al. 2009). The majority of the N applied as chemical fertilizers is lost by evaporation, lixiviation, or volatilization, resulting in soil and water pollution and health problems (Hakeem et al. 2017; Nadarajan and Sukumaran 2021). Therefore, the development of novel biotechnologies that enhance plant N use efficiency (NUE) to enable crop rentability while reducing the environmental impact is a major goal to attain a sustainable agriculture (Sainju et al. 2020; Liu et al. 2022). In this scenario, microbial inoculants that increase the nutrients use efficiency of plants are a promising technology in the agricultural business, as more consumers search for eco-friendly products (Di Benedetto et al. 2017; Santos et al. 2019; Basu et al. 2021).

Inoculants are composed of one or a set of microorganisms capable of stimulating plant development and health (Santos et al. 2019). Among these beneficial microorganisms, plant growth–promoting rhizobacteria (PGPR) are of particular interest due to their phytostimulation, biofertilization, or biocontrol mechanisms (Liu et al. 2022). Within this group, fluorescent *Pseudomonas* outstand as biocontrol agents through the production of secondary metabolites that antagonize phytopathogens (Haas and Défago 2005; Ganeshan and Kumar 2005; Guo et al. 2020), release organic acids to solubilize phosphates (Park et al. 2009), promote the growth of plants by producing phytohormones (Maheshwari et al. 2015), and improve plant tolerance to different stresses (Upadhyay and Srivastava 2014; Banaei-Asl et al. 2016; Pravisya et al. 2019).

Nitric oxide synthase (NOS) is an enzyme that catalyzes the oxidation of l-arginine (Arg) to l-citrulline and nitric oxide (NO). The NOS from the cyanobacteria *Synechococcus* sp. PCC 7335 (SyNOS) is an atypical NOS in that it not only contains oxygenase (NOS_ox_) and reductase (NOS_red_) domains, but also includes a C-terminal globin (NOS_g_) domain (Correa-Aragunde et al. 2018). It has been postulated that SyNOS enzyme first transforms l-Arg to NO through NOS_ox_ and then, most of the NO (ca. 75%) is converted to NO_3_^−^ by NOS_g_, which in turn might be assimilated into amino acid biosynthesis pathways (Picciano and Crane 2020). Recombinant SyNOS enzyme expressed in *Escherichia coli* was demonstrated to be active, resulting in higher bacterial growth rate under N deficiency (Correa-Aragunde et al. 2018).

Given the characteristics of this enzyme, we hypothesized that, if expressed in a PGPR strain, SyNOS enzyme may offer a dual benefit: the residual NO could act as a signal molecule for the bacteria, promoting biofilm formation and, in turn, root colonization (Arruebarrena Di Palma et al. 2013; Vaishnav et al. 2016; Kang et al. 2022). But also, when interacting with plants, SyNOS-expressing bacteria may induce lateral and adventitious root development by NO release (Lamattina et al. 2003). In addition, bacteria expressing SyNOS enzyme could provide the plant with additional NO_3_^−^ to be assimilated in the amino acid biosynthesis pathways. This last process could be a valid strategy to improve N use efficiency (del Castello et al. 2020; Kang et al. 2022).

*Brachypodium distachyon*, commonly called purple false brome, is a monocotyledonous plant of small stature, ca. 15 cm, with a typical generation time of 3 months that has been proposed as a model of C3 plants due to its simple growth conditions in chambers, a completely sequenced genome, and a collection of genetic mutants available. In addition, the overall genetic similarity between *B. distachyon* and crops of agronomic interest such as wheat, barley, and other winter grasses (Brkljacic et al. 2011; Raissig and Woods 2022) allows the use of this species as a grass research model.

In this work, we explored if the heterologous expression of *synos* gene in the PGPR *Pseudomonas koreensis* MME3 modifies its capacity to promote plant growth and improves the adaptability to low N of inoculated *B. distachyon* plants.

## Material and methods

### Bacterial strains and plasmid constructions

*P. koreensis* MME3 was previously isolated from tomato roots (*Solanum lycopersicum* cv. MoneyMaker) and genotyped by *rpoD* sequencing (Maroniche et al. 2016). The strain MME3 of *P. koreensis* has been deposited within the BPCV collection (World Federation for Culture Collection-WDCM31), access number: LBPCV MME3. Starter cultures of MME3 were grown in nutrient broth (NB, 3 g.L^−1^ meet extract and 5 g.L^−1^ pluripeptone, Laboratorios Britania, Buenos Aires, Argentina) at 28 °C for 18 h with orbital shaking (150 rpm). When required, gentamicin (Gm) or tetracycline (Tc) was added at final concentrations of 20 μg.mL^−1^ or 100 μg.mL^−1^, respectively.

For the heterologous expression of SyNOS enzyme in strain MME3, the miniTn*7* system was used (Bao et al. 1991). To this end, the plasmid pMT7Ga-SyNOS was constructed in several steps. First, the *E. coli* TOP10 (Invitrogen, Waltham, MA, USA) *rrnB* double terminator was excised from pCR8/GW/TOPO (Invitrogen, Waltham, MA, USA) with *Nhe*I and ligated into the *Xba*I site of pME3280a (Zuber et al. 2003), which had been previously modified to eliminate the duplicated *Eco*RI and *Sac*I restriction sites outside the polylinker. Then, the promoter P_A1/O4/O3_ – RBS from pBBR2-PA1 was ligated between *Hin*dIII and *Eco*RI restriction sites, obtaining pMT7Ga. Finally, the *synos* coding sequence was extracted from pET24a-SyNOS (Correa-Aragunde et al. 2018) and inserted into pMT7Ga with *Nsi*I and *Eco*RI. The resulting pMT7Ga-SyNOS suicide plasmid along with the Tn*7* transposition helper pUX-BF13 (Bao et al. 1991) was introduced in *P. koreensis* MME3 by tetraparental mating using the conjugation helper plasmid pRK600 (Finan et al. 1986). Recombinant clones carrying the transposed *synos* expression cassette (MME3:SyNOS) were recovered by plating on nutrient agar (NA), prepared using NB added with 15 g.L^−1^ bacteriological agar (Laboratorios Britania, Buenos Aires, Argentina) containing 20 µg.mL^−1^ of Gm and 20 µg.mL^−1^ of trimethoprim as a counter-selection. The pMT7Ga empty vector was used by the same procedure to obtain a negative control recombinant strain (MME3:c) that contains the gentamicin resistance cassette in its chromosome. Unless stated otherwise, all the enzymes were purchased from New England Biolabs (NEB, Hitchin, UK).

To confirm the correct insertion of miniTn*7* constructs, we performed PCR, using the primers Tn7GlmS (5ʹAATCTGGCCAAGTCGGTGAC3ʹ) and GmR5ʹ RV (5ʹCAAGCCATGAAAACCGCCACT3ʹ), with an annealing temperature of 50 °C for 45 s and extension at 72 °C for 30 s. To corroborate the presence of the *synos* gene, we performed PCR using the primers SyNOSfw (5ʹCGAATTCATGAATGAGCAGGCATGGGAGGAGAGCGATCGCGAGCGCCGCTGGCAAGAG3ʹ) and SyNOSrv (5ʹGCTCGAGTCACAGGTCCTCCTCTGAGATCAGCTTCTGCTCCGTGGTTTGAACAAACTG3ʹ), with an annealing temperature of 55 °C for 30 s and extension at 72 °C for 30 s.

Fluorescent strains were obtained by transforming MME3:c and MME3:SyNOS with plasmid pMP4655 (Tc^R^, P_lac_-*egfp*) (Bloemberg et al. 2000) by triparental mating. Recombinant clones MME3:c:GFP and MME3:SyNOS:GFP were recovered by plating on NA containing Gm and Tc.

### RT-PCR analysis

Total RNA was extracted from MME3:c and MME3:SyNOS with 1 mL of TRI Reagent (MRC, Inc., Cincinnati, OH, USA). The RNA was treated with 1.5 U of DNAase I (Promega, Madison, WI, USA) per µg of RNA, and then used for cDNA synthesis in reactions containing 0.5 µg.µL^−1^ of random primers and 200 U of M-MLV reverse transcriptase (Invitrogen, Waltham, MA, USA). To check for contamination with genomic DNA, PCR was performed using *rpoD* primers PsEG30F (5ʹATYGAAATCGCCAARCG3ʹ) and PsEG790R (5ʹCGGTTGATKTCCTTGA3ʹ) (Mulet et al. 2009) using RNA extracts as template. Analysis of the *synos* gene expression in the recombinant strains was done with primers rtSyNOSfw (5ʹTCGGCAGCACCGTCTATGAA3ʹ) and rtSyNOSrv (5ʹTCGGCTTGTCCTTTGATTTCG3ʹ).

### Growth curves

Aliquots of 600 µL from starter cultures were used to inoculate Erlenmeyer flasks containing 30 mL of NB supplemented with 5 mM l-Arg. The cultures were grown at 28 °C with orbital agitation at 150 rpm. Growth was followed by optical density at 600 nm (OD_600nm_) measured with a spectrophotometer (SmartSpec3000, BioRad, Hercules, CA, USA), until the stationary phase and then at 48 h in the late stationary phase. The curves were fitted to sigmoidal models with Prism 8 (GraphPad Software Inc., La Jolla, CA, USA). The average of growth rate (*µ*) was calculated as the slope at a straight-line tangent to the growth curve in the exponential phase.

### Quantification of nitrate and protein content

Cultures were obtained as explained above. After centrifugation at 6000 rpm for 15 min, the supernatants were collected and cells re-suspended in phosphate-buffered saline (PBS, 0.1 M, pH = 7). The final OD_600nm_ was measured and adjusted to 6. The bacteria in the suspensions were lysed by sonication (four rounds of four cycles for 20 s each, on ice, amplitude 100%, Sonic Vibra-Cell, Sonics and Materials INC., Newtown, CT, USA) and centrifuged at 6000 rpm and 4 °C for 10 min to discard cell debris. The supernatants were lyophilized (model LA-B4, Rificor, Buenos Aires, Argentina) and re-suspended in PBS to concentrate them 10 × . NO_2_^−^ and NO_3_^−^ contents were measured by the Griess method (Miranda et al. 2001). For NO_2_^−^ measurement, 50 μL of bacterial suspensions or supernatants was mixed in 96-well microplates with 50 μL of 2% sulfanilamide (SA) in 5% H_3_PO_4_ and 50 μL of 0.1% *N*-(1-naphthyl) ethylenediamine dihydrochloride (NED) and incubated for 10 min in the dark. The absorbance at 540 nm was measured using an Epoch Microplate Spectrophotometer (BioTek, Winooski, VT, USA). For NO_3_^−^ content determination, total NO_3_^−^ in samples was completely reduced to nitrite with 50 μL of 0.8% vanadium (III) chloride in 1 M HCl; then, SA and NED were added and incubated during 30 min at 37 °C (Miranda et al. 2001). Protein content in samples was measured by the Bradford method (Bradford 1976). All drugs were purchased from Sigma-Aldrich, St. Louis, MO, USA.

### Phenotypic characterization of *P. koreensis* MME3:SyNOS

Starter cultures in test tubes were centrifuged at 6000 rpm for 6 min, and cells were re-suspended in sterile saline solution (SS; 0.85% NaCl) to a final OD_600nm_ = 1 (containing approximately 10^9^ colony forming units (CFU). mL^−1^). Drops of 10 μL of bacterial suspensions were inoculated on King’s B medium (King et al. 1954) with or without 5 mM l-Arg. After incubating the plates for 48 h at 28 °C, siderophore production was revealed by the universal chrome azurol S (O-CAS) assay (Pérez-Miranda et al. 2007).

Swarming assay was performed in NA plates with 0.5% agar and 0.5% glucose, with or without 5 mM l-Arg. Drops of 10 μL of bacterial suspensions were inoculated, plates were incubated for 48 h at 28 °C, and then, the swarming area was measured. Swimming was estimated measuring halo produced in NA medium containing 0.3% agar, with or without 5 mM l-Arg. In this case, suspensions were inoculated with a sterile toothpick and plates were incubated as before. Images were processed with ImageJ software (https://imagej.nih.gov/ij/).

To estimate auxin production, Erlenmeyer flasks containing 30 mL of NB + 5 mM l-Arg, supplemented with or without 5 mM l-tryptophan, were inoculated with 600 µL of bacterial suspensions of OD_600nm_ = 1, and incubated at 28 °C with agitation at 150 rpm. After 18 h, cultures were centrifugated and 1 mL of supernatant was collected and incubated with 1 mM sulfamic acid for 1 h to prevent nitrate from destroying indole-3-acetic acid (IAA) in acid solution. Then, IAA content was estimated by the Salkowski method (Glickmann and Dessaux 1995).

### Biofilm formation

Biofilm formation was analyzed in NB and Luria–Bertani media (LB, 10 g.L^−1^ tryptone, 5 g.L^−1^ yeast extract, and 10 g.L^−1^ NaCl, pH 7) (Bertani 1951), with or without 5 mM l-Arg by the crystal violet method (O’Toole and Kolter 1998). Briefly, 200 µL of medium containing 10^7^ CFU of MME3:c or MME3:SyNOS was seeded into polystyrene microtiter 96-well flat bottom plates and incubated under static conditions at 28 °C for 48 h. Biofilms formed in polystyrene paralleled wells were quantified by crystal violet dye staining followed by colorimetry (OD_550nm_), and normalized by total growth (OD_600nm_). Total growth quantification OD_600nm_ was registered after mechanical disaggregation and mixed with planktonic cells (Arruebarrena Di Palma et al. 2013). In parallel, the number of adhered cells in the biofilm was quantified using fluorescent strains. To that end, biofilm formation was performed in the same way as described above but MME3:c:GFP and MME3:SyNOS:GFP were used instead. The number of adhered cells and total growth were estimated by measuring fluorescence intensity (excitation 480 nm; emission 528 nm) with a fluorescent microplate reader (Fluoroskan Ascent; Thermo Electron, Waltham, MA, USA).

### Growth promotion of *B. distachyon* in semi-hydroponic culture

Seeds of *B. distachyon* (cv. Bd21-3) were surface-sterilized by immersion in 70% ethanol for 30 s, and then rinsed three times with sterile distilled water, immersed in 1.3% sodium hypochlorite for 4 min, and rinsed again. Disinfected seeds were settled on top of sterile filter paper imbibed with sterile distilled water in a Petri dish. After incubation for 4 days in the dark at 4 °C to synchronize germination, plates were placed in a germination chamber at 20 °C with a 16/8-h light/dark photoperiod. Twenty-five seedlings of 7 days old per treatment were transferred to 400 cm^3^ pots containing sterile sand (one seedling per pot) and immediately inoculated at the base of the stem with 100 µL of bacterial suspensions of MME3:c or MME3:SyNOS (DO_600nm_ = 1, containing ca. 10^9^ CFU.mL^−1^), and raised in a growth chamber at 25 °C under a photoperiod of 16 h. Non-inoculated seedlings (ni) were used as control. Plants were watered once a week with ATS nutrient solution (2 mM CaSO_4_.2H_2_O, 2 mM MgSO_4_.7H_2_O, 2.5 mM KH_2_PO_4_, 50 µM Fe-EDTA and micronutrients: 70 µM H_3_BO_3_, 14 µM MnCl_2_, 1 µM ZnSO_4_, 0.5 µM CuSO_4_, 0.2 μM Na_2_MoO_4_, 10 μM NaCl, 0.01 μM CoCl_2_). N content in ATS was 9 mM KNO_3_ for N sufficiency (high N semi-hydroponic, HN-Hy) or 1 mM KNO_3_ for N deficiency (low N semi-hydroponic, LN-Hy). In the LN-Hy condition, K was compensated by adding 4 mM of KCl. After 35 days post-inoculation (dpi), 15 plants per treatment were harvested to measure shoot fresh weight and shoot and root dry weights. These tissues were dried in an oven at 60 °C until reaching constant weight. The chlorophyll and carotenoid contents from the whole aerial part of five plants were measured after extraction with 100% cold ethanol (0.45 g of fresh tissue in 10 mL solution) at 4 °C in the darkness for 24 h. Total chlorophyll content was calculated measuring the absorbance at 470, 648, and 664 nm and using the Lichtenthaler equations (Lichtenthaler 1987). The whole aerial part of the other five plants were used to measure NO_3_^−^ content by the Griess method (Miranda et al. 2001).

### Growth promotion of *B. distachyon* in the greenhouse

Seeds of *B. distachyon* (cv. Bd21-3) were sterilized and treated as describe above. Thirty 7-day-old seedlings per treatment were transferred to 2-L pots containing mixtures of soil:sand:perlite at ratios of 1:1:1 or 1:4:4 for growth in high N soil (HN-S) or low N soil (LN-S), respectively. Plants were inoculated as explained before (MME3:c, MME3:SyNOS), or not inoculated (control treatment: ni), and raised in a greenhouse during 90 days in winter season under natural light, in Balcarce, province of Buenos Aires, Argentina (south latitude 37°50′47″ and west longitude 58°15′20″). Plants were watered weekly with distilled water. Forty and 60 dpi plants were watered with ATS containing 1 mM KNO_3_ or 9 mM KNO_3_ for LN-S and HN-S conditions, respectively. At 35 dpi, 15 plants were harvested and 10 of them were used for measurement of shoot fresh weight, and shoot and root dry weights. The chlorophyll and carotenoid contents from the whole aerial part of five plants were measured (Lichtenthaler 1987). The rest of the plants were maintained in the greenhouse until the spikes reached physiological maturity. In those plants, shoot dry weight, leaves’ total N content, and grain yield were measured.

Soil and grain total N contents were determined by the Kjeldahl method (Nelson and Sommers 1973). The N uptake efficiency (NUpE), defined as the quantity of resource absorbed with respect to its availability, was estimated by Eq. (1), described as the ratio of N in grain with respect to N available (Zhang et al. 2015), where N available is the sum of N in soil at the start of experiment plus N supplied by the watering with ATS, N content remaining at the end of experiment, and N in plant (adapted from Dawson et al. 2008; Moll et al. 1982).

1 $${\mathrm{NUpE}}=\frac{\mathrm{N\; grain}}{\mathrm{N\; available}}$$

The N use efficiency (NUE), described as the ratio of yield respect to N available, was calculated by Eq. (2), where yield is the total grain weight per plant and N available as explained above (adapted from Dawson et al. 2008; Dobermann 2007).

2 $${\mathrm{NUE}}=\frac{{\mathrm{Yield}}}{\mathrm{N \;available}}$$

### Experimental design and statistical analysis

For growth kinetics studies, each condition was analyzed with four independent biological replicas with two technical repetitions each. Assays for phenotypic studies were done with four independent biological replicas and four repetitions for each measured variable, as indicated in the figure legends. ANOVA was performed and the normality of the residuals and homoscedasticity of the variance were assessed with Shapiro–Wilk and Bartlett tests. Media were compared using *t*-test (*p* < 0.05 or *p* < 0.1, as indicated in each case in figure legends). Data analysis was accomplished with Prism 8 (GraphPad Software Inc., La Jolla, CA, USA). Results were expressed as means ± standard error (SE).

For sand-based semi-hydroponic assay, a randomized complete design was arranged for each N condition. The three treatment set ups were as follows: (i) not inoculated control (ni), (ii) inoculated with MME3:c, (iii) inoculated with MME3:SyNOS. Each treatment consisted of 15 plants used to measure fresh and dry weights of shoots, and dry weight of roots. One-way ANOVA was performed using the statistical software Prism 8. Media were compared using Tukey’s post-test or Sidak’s multiple comparison post-test as appropriate.

The experimental design used in greenhouse trial for each N condition was a completely randomized block design with three blocks and three treatments as described above. Each treatment sample consisted of 10 plants in each of the three blocks. Analysis of variance was performed using the statistical software R (version 4.2.1, R Foundation for Statistical Computing, Vienna, Austria). The normality of the residuals and homoscedasticity of the variance were assessed with Shapiro–Wilk and Levene tests. Media were compared using Tukey’s post-test. Differences were considered to be significant at *p* < 0.05 (indicated by two asterisks) or *p* < 0.1 (indicated by one asterisk).

## Results

### Effect of the expression of the NO synthase SyNOS on *P. koreensis* MME3 growth

**Fig. 1:**
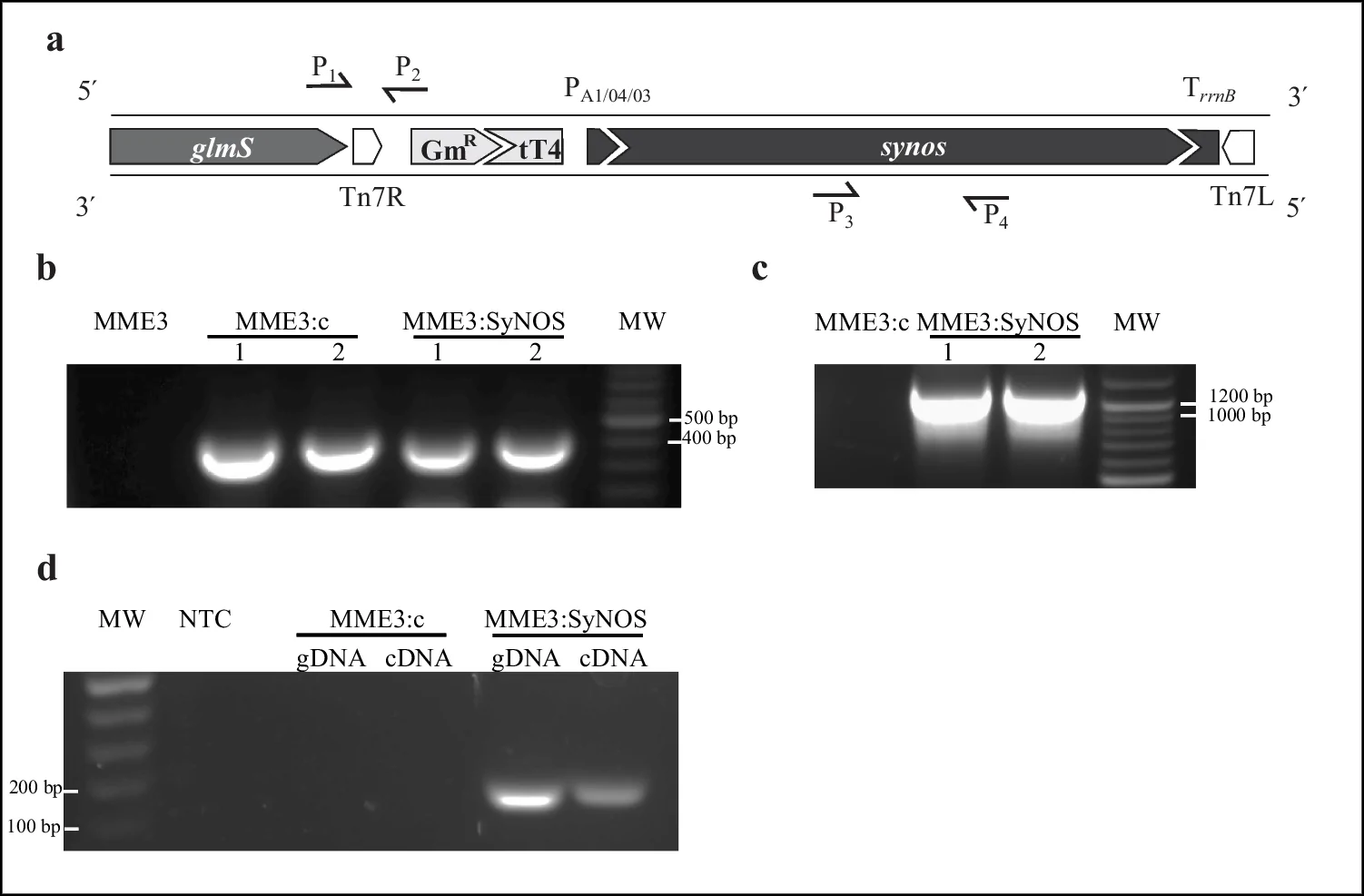
Genetic modification of *P. koreensis* MME3 to express *synos*. **a** Schematic representation of the miniTn*7* carrying a *synos* expression cassette, inserted into the bacterial chromosome downstream of the *glmS* gene. GmR cassette confers resistance to gentamicin. The *synos* cassette is composed of the P_A1/04/03_ promoter, the *synos* coding sequence, and the T_*rrnB*_ terminator. The half black arrows represent primers used to confirm the correct insertion of the miniTn*7* (P_1_ = Tn7GlmS and P_2_ = GmR5ʹ RV) and the presence of *synos* (P_3_ = SyNOSfw and P_4_ = SyNOSrv). **b** Agarose gel electrophoresis of PCR products obtained by amplification with primers P_1_ and P_2_, showing the expected product of 442 bp, from two clones carrying the empty miniTn*7*(GmR) transposon (MME3:c1 and MME3:c2) as well as two clones carrying the *synos* miniTn*7*(GmR + SyNOS) transposon (MME3:SyNOS.1 and MME3:SyNOS.2). DNA ladder (100 bp) was included to determine product size (MW). **c** Agarose gel electrophoresis revealing partial PCR amplification of *synos* coding sequence with primers P_3_ and P_4_, showing the expected product of 1136 bp, from the recombinant clones. **d** Agarose gel electrophoresis of RT-PCR products obtained by partial amplification of *synos* transcript from MME3:SyNOS and MME3:c control strain. Total RNA extracts were used as template for RT-PCR using primers rtSyNOSfw and rtSyNOSrv (expected product of 178 bp)

A variant of *P. koreensis* MME3 that expresses *synos*, a NO synthase gene from the cyanobacterium *Synechococcus* sp. PCC 7335, was developed. A miniTn*7* transposon that carries a *synos* expression cassette, along with a gentamicin resistance determinant, was constructed and inserted into *P. koreensis* MME3 chromosome as a single copy, to obtain strain MME3:SyNOS (Fig. 1a). A gentamicin-resistant control strain (MME3:c) was also obtained by using the unmodified transposon. Genomic PCR analyses of two independent clones confirmed the correct insertion of the transposons in both MME3:c and MME3:SyNOS genomes (Fig. 1b) and the presence of the *synos* gene in MME3:SyNOS (Fig. 1c). The expression of *synos* was verified by detecting the transcript in MME3:SyNOS via RT-PCR (Fig. 1d).

**Fig. 2:**
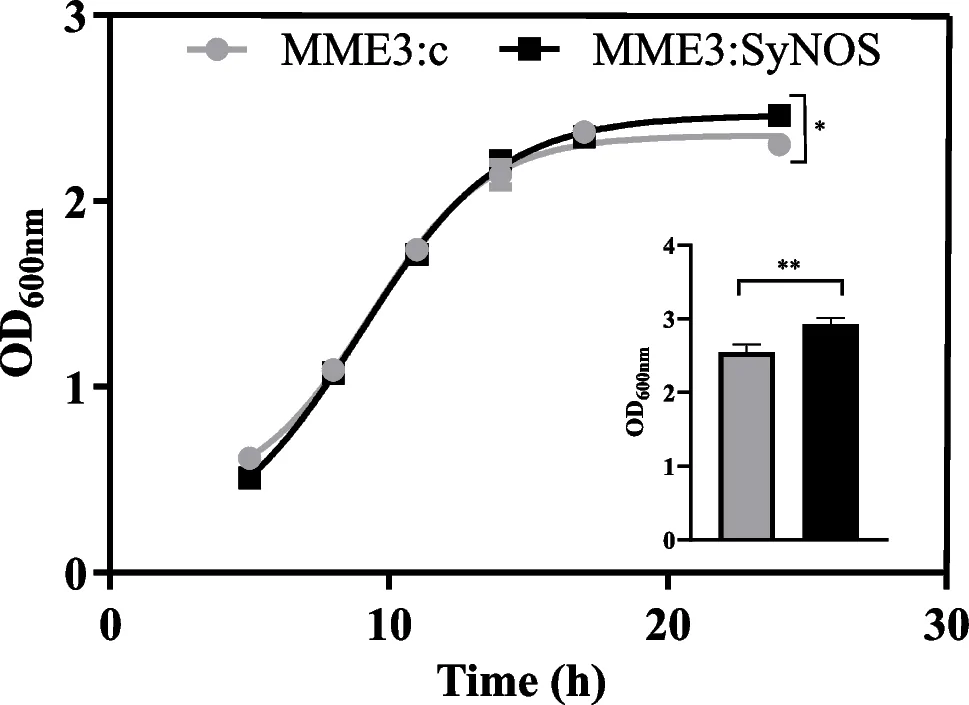
Growth kinetics of MME3:SyNOS and MME3:c (control) strains. Starter cultures grown in NB overnight were used to inoculate fresh NB + 5 mM l-Arg. MME3:c (gray circles) and MME3:SyNOS (black squares) were cultured under 150 rpm agitation at 28 °C. The OD at 600 nm was measured following the growth until reaching the stationary phase. The inset shows measurements done at the late stationary phase (48 h). For each condition, four independent replicas were used. Plotted values are means ± SE. Asterisks indicate statistically significant differences with a significance of *p* < 0.1 (*) or *p* < 0.05 (**) according to *t*-test analyses

We investigated the effect of SyNOS expression on bacterial growth in NB supplemented with arginine to ensure that the enzyme had all the factors necessary to function properly. The growth rate *µ* for MME3:SyNOS was 0.192 (± 0.007) and significantly higher (*p* < 0.05) than the growth rate for MME3:c (*µ* = 0.174, ± 0.011). The absorbance measured after 24 h of growth was significantly higher in MME3:SyNOS when compared to that in MME3:c (*p* < 0.1), and this difference increased at 48 h of growth (*p* < 0.05, inset in Fig. 2). This result suggested that the presence of SyNOS enzyme improves bacterial development during the stationary phase (Fig. 2).

Since fluorescent *Pseudomonas* are able to use nitrate as alternative electron acceptor under oxygen limitation (Redondo-Nieto et al. 2013; Tang et al. 2020), as occurs during the stationary phase, we measured the intracellular and extracellular nitrate contents of the cultures at the stationary phase. Though not statistical significant, both the pellet and the supernatant of MME3:SyNOS had a higher mean content of nitrate (Table 1). The sum of nitrate content in the pellet and supernatant was, in average, 11.5% higher in MME3:SyNOS than in MME3:c. The protein did not differ significantly, although content by SyNOS enzyme expression was 9.1% higher than in the control strain (Table 1).

**Table 1:** NO_3_^−^ and total proteins content in MME3:SyNOS or MME3:c grown in NB medium + 5 mM l-Arg

	NO_3_^−^ (µM)		Proteins (µg.mL^−1^)
	Supernatant	Pellet	Total
MME3:c	6.48 ^a^ ± 0.28	17.12 ^a^ ± 0.42	156.51 ^a^ ± 7.63
MME3:SyNOS	7.68 ^a^ ± 0.87	18.73 ^a^ ± 0.57	170.75 ^a^ ± 5.32

^a^Letters indicate not significant differences between both strains for each dataset analyzed according to *t*-test

### Expression of SyNOS enzyme modifies *P. koreensis* MME3 traits important for rhizospheric colonization

**Fig. 3:**
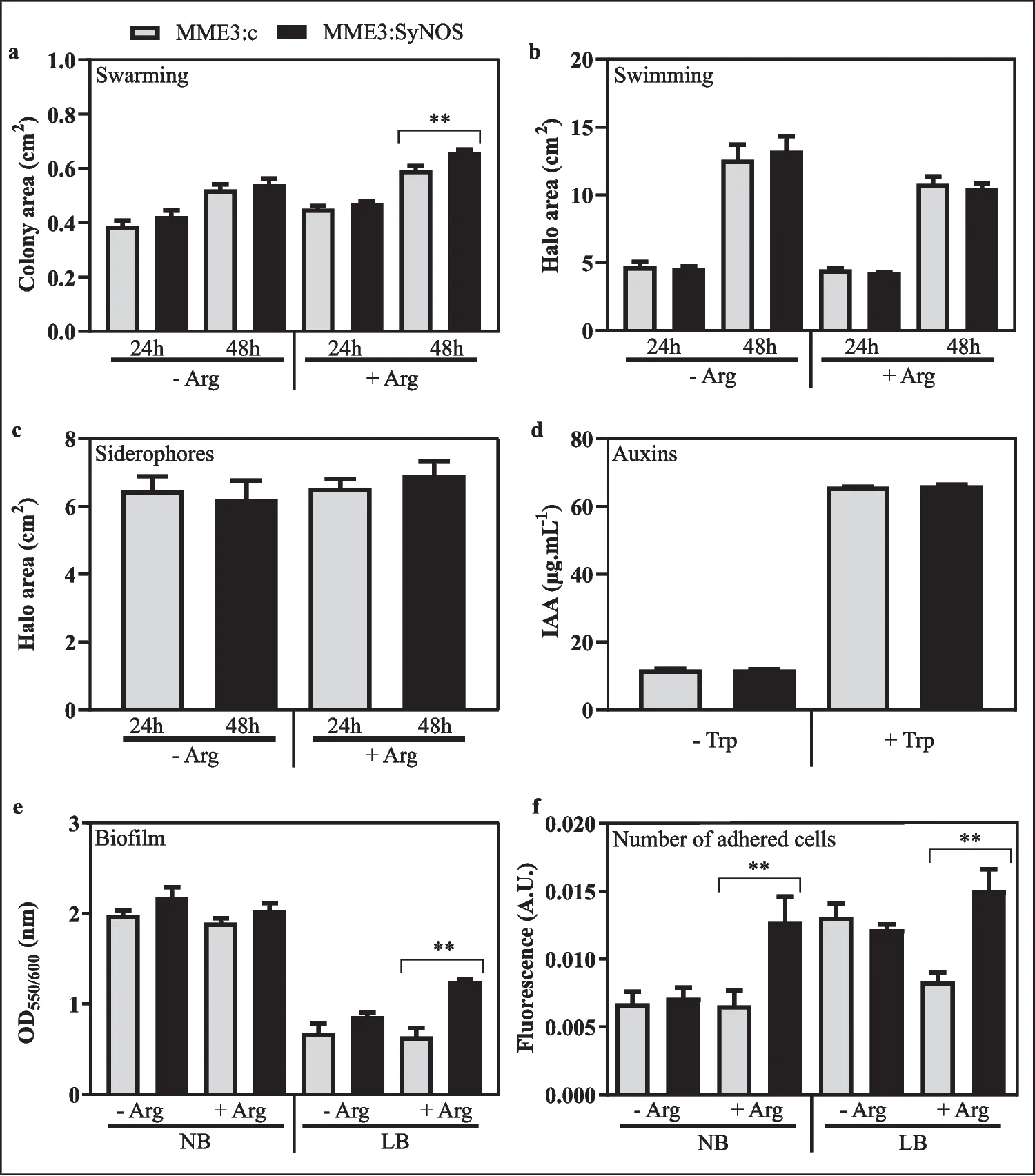
Phenotypic characterization of strain MME3:SyNOS. Bacterial cultures of MME3:SyNOS and MME3:c were grown overnight in NB medium. Aliquots of 10 μL of bacterial suspensions containing approximately 10^9^ CFU.mL^−1^ were used in each assay. Swarming motility, measured from the area of colony growth on NA medium containing 0.5% (w/v) agar + 0.5% (w/v) glucose with or without 5 mM l-Arg (**a**). Swimming motility, measured in the same way, on NA medium with 0.3% (w/v) agar with or without 5 mM l-Arg (**b**). Siderophore production in King’s B with or without 5 mM l-Arg was measured from the orange halos, as revealed by the O-CAS assay. In all cases, the halos or colony areas were determined with ImageJ software (**c**). Production of IAA in NB medium containing 5 mM l-Arg with or without 5 mM l-Trp as estimated by the Salkowski method (**d**). In vitro biofilm formation in static cultures in NB or LB media, with or without 5 mM l-Arg. Approximately 10^7^ CFU.mL.^−1^ of MME3:c and MME3:SyNOS was seeded in 96-well plates and cultured for 2 days at 28 °C under static conditions. Biofilm formation was determined by the crystal violet method measuring OD at 550 nm and normalizing data by the total growth (OD_550_/OD_600_) (**e**). The number of adhered cells was analyzed by measuring the fluorescence intensity (excitation 480 nm; emission 528 nm) of the cells present in biofilms formed by MME3:c:GFP and MME3:SyNOS:GFP (A.U., arbitrary fluorescence units) (**f**). Plotted values are the average of four independent replicas. Plotted values are means ± SE. **Statistically significant differences (*t*-test, *p* < 0.05)

We analyzed if SyNOS expression altered MME3 physiological processes important for a successful establishment in the rhizosphere and plant growth promotion (Knights et al. 2021). We studied the swarming and swimming motilities in semisolid NA medium. After 48 h of growth, swarming of MME3:SyNOS was higher (*p* < 0.05) than in the control strain, as evidenced by an increased expansion of macrocolonies in the media (Fig. 3a). On the contrary, swimming motility was unmodified by the expression of SyNOS (Fig. 3b).

We also tested the production of siderophores, since it is a valuable trait for a PGPR and a distinctive characteristic of fluorescent *Pseudomonas* (Ghazy and El-Nahrawy 2021). The halos exposed by the O-CAS assay did not differ significantly between strains, although MME3:SyNOS halo had a slightly larger area than that produced by MME3:c in the presence of arginine (Fig. 3c).

The production and secretion of auxins were tested, since it is one of the most researched mechanisms underlying PGPR action on plants (Guo et al. 2021). It was observed that the production of auxinic compounds, both in the absence or the presence of the precursor tryptophan, was similar in both MME3:c and MME3:SyNOS (Fig. 3d).

The biofilm formation and cell adherence on abiotic surfaces were tested in both NB and LB media. MME3:SyNOS showed an overall tendency to form more biofilm when compared with MME3:c, although this tendency was significant only in LB + arginine medium (*p* < 0.05) (Fig. 3e). Besides, in both NB and LB media supplemented with arginine, a significantly greater fluorescence (*p* < 0.05) was detected in the biofilm formed by MME3:SyNOS:GFP, indicating higher number of cells adhered to the surface (Fig. 3f).

### Bacteria improve plant growth under nitrogen starvation

**Fig. 4:**
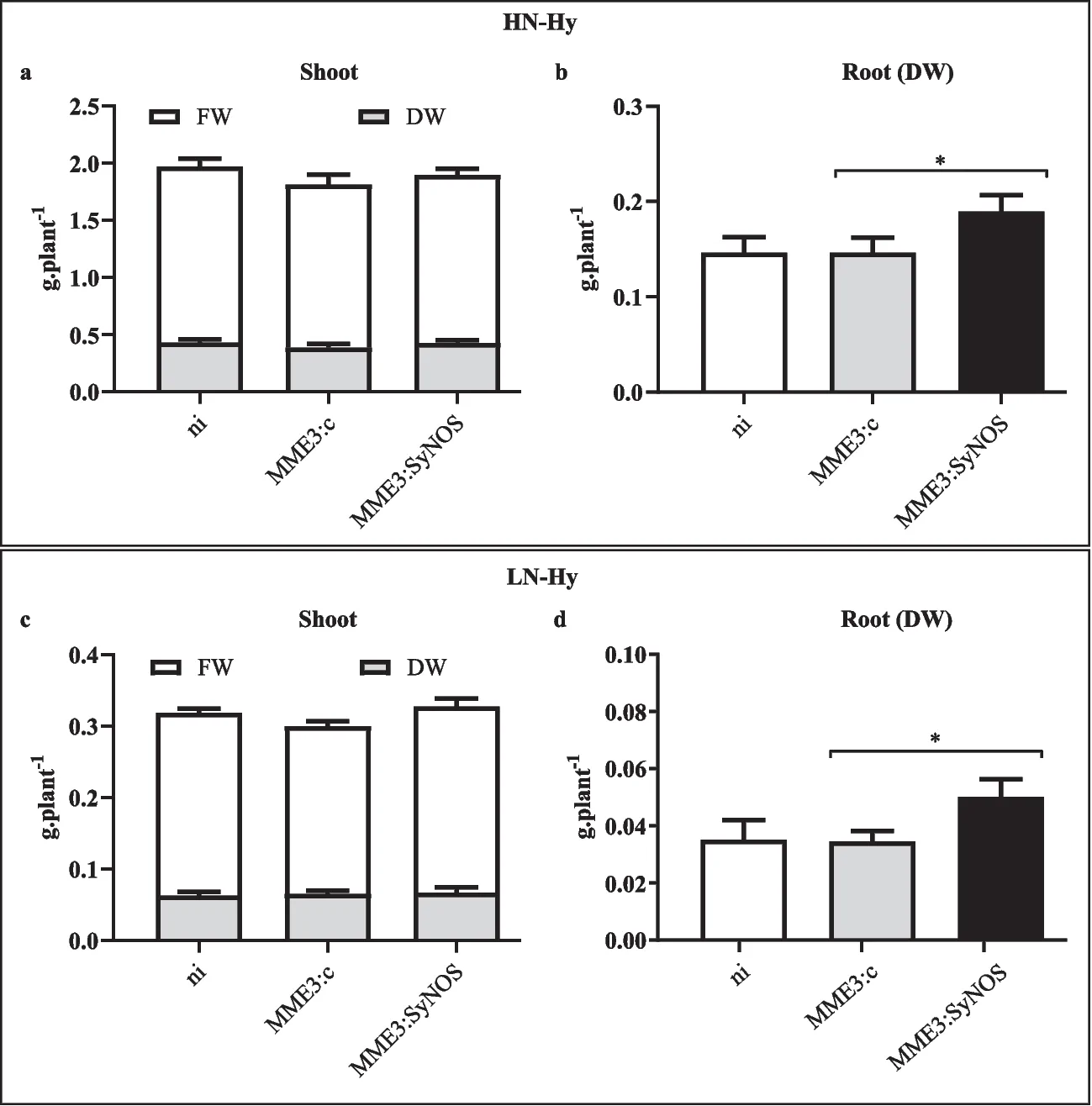
Effect of inoculation on *Brachypodium distachyon* with *P. koreensis* MME3:SyNOS grown under semi-hydroponics in sand. Seeds of *B. distachyon* were surface-sterilized and stratified. The plates were placed in a germination chamber at 20 °C with a 16/8-h light/dark photoperiod. Seven-day-old seedlings were transferred to 400 cm^3^ pots containing sterile sand and inoculated with 100 µL of MME3:c or MME3:SyNOS bacterial suspension. The plants were grown semi-hydroponically in a chamber under a photoperiod of 16 h of light at 25 °C. Non-inoculated seedlings (ni) were used as a control. Plants were watered once a week with ATS containing high nitrogen (HN-Hy, 9 mM KNO_3_) (**a**, **b**) or low nitrogen (LN-Hy, 1 mM KNO_3_) concentrations (**c**, **d**). After 35 days, plants were harvested and fresh weights (FW) and dry weights (DW) of the shoot and DW of root were measured. Plotted values are the average of 15 independent plants ± SE. Results were statistically analyzed by ANOVA and Sidak’s multiple comparison post-test. *Statistically significant differences (*p* < 0.1)

*B. distachyon* was selected as an experimental model of C3 grasses to study the plant growth promotion capacity of MME3:SyNOS strain. *B. distachyon* was inoculated with MME3:SyNOS or MME3:c or not inoculated (ni), and grown in sand-based semi-hydroponic culture for 35 days, watering with ATS containing 9 mM KNO_3_ (HN-Hy) or 1 mM KNO_3_ (LN-Hy). When the chlorophyll, carotenoid, and nitrate contents in aerial organs were analyzed, no significant differences were observed between treatments for any of the measured parameters (data not shown). Also, no significant differences were observed in fresh and dry shoot weighs (Fig. 4a, c). However, MME3:SyNOS-inoculated plants showed significant higher root dry weight (*p* < 0.08) when grown in HN-Hy, producing 30% more dry matter than MME3:c plants (Fig. 4b). MME3:SyNOS inoculation also produced a statistical significant increase (*p* < 0.06) in root dry weight in LN-Hy, resulting in 43% more dry mater than MME3:c-inoculated plants (Fig. 4d). These results revealed that inoculation with MME3:SyNOS strain promotes root growth.

**Fig. 5:**
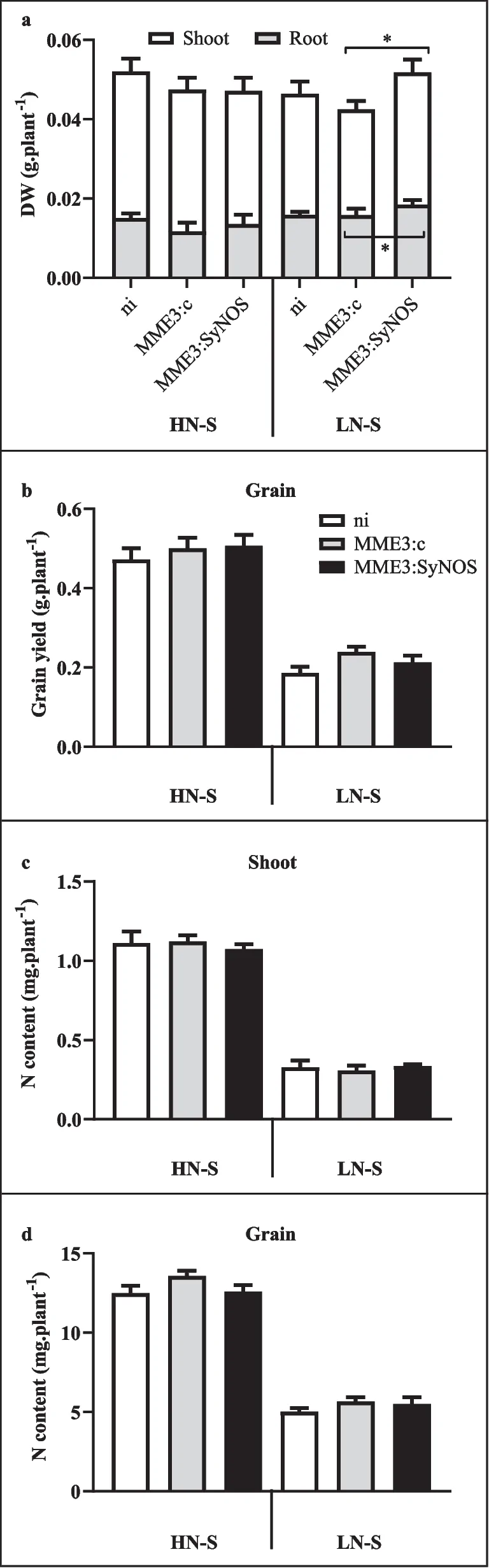
Effect of inoculation on *B. distachyon* with *P. koreensis* MME3:SyNOS grown in a greenhouse under different soil conditions. Seeds of *B. distachyon* were surface-sterilized and stratified. Seedlings were transferred to 2-L pots containing high N soil (HN-S) or low N soil (LN-S) consisting of mixtures of soil:sand:perlite in 1:1:1 or 1:4:4 ratios, respectively, and inoculated with 100 µL of MME3:c or MME3:SyNOS bacterial suspension. Non-inoculated (ni) seedlings were used as a control. Plants were grown in a greenhouse in winter. **a** After 35 days, half of the plants were harvested and dry weights of the shoot and root parts were measured. Plotted values are the average of five replicas in each of three blocks ± SE. The other 15 plants were harvested when the spikes were mature to analyze grain yield (**b**), and total N content in shoots (**c**) and grains (**d**). Results were statistically analyzed by ANOVA and Turkey’s test. *Statistically significant differences (*p* < 0.1)

Finally, in order to get closer to field conditions, we grew *B. distachyon* plants in a greenhouse during winter season under HN-S or LN-S conditions generated by two different soil compositions. Plants were sampled at 35 (vegetative growth) and 90 dpi (harvest time). At 35 dpi under LN-S, MME3:SyNOS-inoculated plants showed significantly higher shoot (*p* < 0.1) and root dry weights (*p* < 0.08) (Fig. 5a). Although grain yield differences were not statistically significant, inoculation treatments increased the average yield in 6% for MME3:c and 7.2% for MME3:SyNOS under HN-S, and 29% for MME3:c and 14% for MME3:SyNOS under LN-S, with respect to non-inoculated treatments (Fig. 5b). We did not observe significant differences in the shoot or grain nitrogen content between any of the conditions (Fig. 5c, d).

**Fig. 6:**
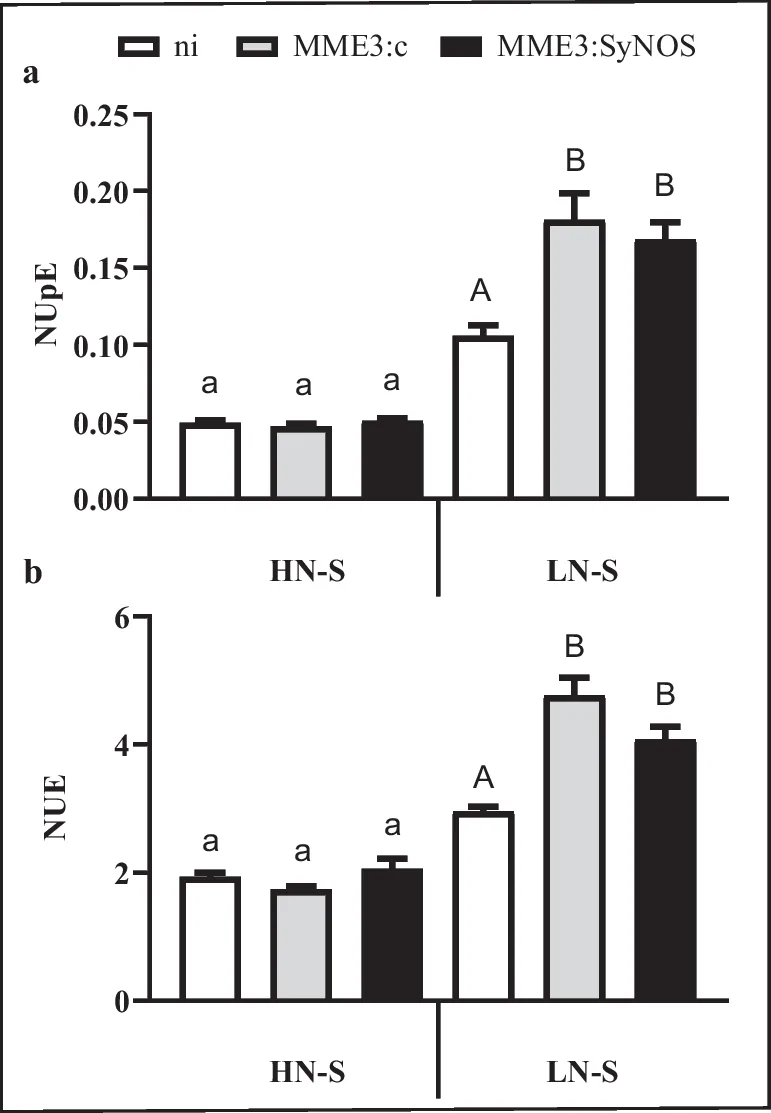
Effect of inoculation with *P. koreensis* MME3:SyNOS on N uptake and use efficiencies of *B. distachyon* grown in a greenhouse under different soil conditions. The plants were growth on high N soil (HN-S) or low N soil (LN-S), consisting of a mixture of soil:sand:perlite in 1:1:1 or 1:4:4 ratios, respectively, and inoculated with 100 µL of MME3:c or MME3:SyNOS bacterial suspension. Seedlings non-inoculated (ni) were used as a control. The N indexes were calculated from N content measurements at harvest time, when the spikes were mature. **a** N uptake efficiency (NUpE) was calculated as *N*_grain_ / *N*_available_, where *N* available is the sum of all N sources measured. **b** N use efficiency (NUE) was calculated as yield / *N*_available_. Plotted values are the average of five replicas in each of three blocks ± SE. Results were statistically analyzed by one-way ANOVA for each condition and Tukey’s test. Different letters indicate significant differences (*p* < 0.05)

The NUpE and NUE indexes did not vary between treatments under HN-S condition (Fig. 6). However, under LN-S, both NUpE and NUE were higher in inoculated plants with MME3:c and MME3:SyNOS (*p* < 0.01 in all cases) (Fig. 6a, b).

## Discussion

MME3 is an endophytic bacterial strain that was isolated from roots of tomato cultivated in lateritic red soil from Misiones province, Argentina (Maroniche et al. 2016). It was shown to possess several characteristics associated to plant growth promotion (PGP), such as phosphate solubilization, and auxins, siderophores, and HCN secretion, the latter being a common biocontrol factor. It also showed in vitro antagonistic activity against *Sclerotinia sclerotiorum* (Maroniche et al. 2016). In fact, the promotion of plant growth by this bacterium was demonstrated in lettuce plants (Maroniche et al. 2016). In this work, we selected strain MME3 as a PGPR model for the heterologous expression of *synos* in an attempt to enhance its PGP capacity (Creus et al. 2005; Molina-Favero et al. 2008). We selected the miniTn*7* system to express the *synos* gene because it allows stable expression of foreign genes by insertion of a unique copy in a neutral position of the chromosome of *Pseudomonas* and other bacterial groups (Koch et al. 2001). This low-level expression strategy was selected also to avoid abrupt pleiotropic effects induced by the alteration of bacterial N metabolism. We then investigated the possibility of improving nitrate assimilation by inoculation with the *synos*-expressing bacteria, which in turn would lead to higher root development and better adaptation to low N availability, as occurs in low input agronomic practices or N-deficient soils (Del Castello et al. 2021).

The replication ability is a crucial factor for inoculant production. Correa-Aragunde et al. (2018) showed that *E. coli* transformed with the *synos* gene improved its growth rate in medium supplemented with arginine. We showed that *synos* expression in MME3:SyNOS improved the bacterial growth rate in nutrient broth supplemented with the substrate l-Arg and achieved higher growth at the stationary growth phase. This could have been due to the use of the accumulated nitrate that can be used as final electron acceptor. Contrary to the control strain, MME3:SyNOS may have an enhanced capacity of aerobic nitrate respiration by the action of globin domain of SyNOS, obtaining nitrate from NO and increasing its growth at final stages of growth curve where oxygen is limited (Chaudhari et al. 2017; Correa-Aragunde et al. 2018). Under this condition, although not statistically different, MME3:SyNOS accumulated 12% more nitrate than control strain. In the rhizosphere, when nitrate availability is limiting, nitrate production could improve the fitness of MME3:SyNOS. In support of this hypothesis, Muriel et al. (2015) showed that the metabolic versatility of fluorescent *Pseudomonas*, specifically their ability to use nitrate under anaerobic conditions as a final electron acceptor, provides an advantage for competition and colonization of the rhizosphere. Moreover, there is evidence of a positive correlation between denitrifying capacity of fluorescent *Pseudomonas* and their rhizosphere colonization capacity in nitrate-supplemented media (Ghiglione et al. 2000; Zboralski and Filion 2020).

Upon characterization of the *P. koreensis* MME3:SyNOS phenotype, we established that *synos* expression induces physiological changes that might alter its association with plants. We demonstrated here that MME3:SyNOS has a greater swarming motility in vitro. It has been shown that bacteria with high motility can access the root more efficiently than bacteria with less motility, which determines the distribution of cells along the root (Capdevila et al. 2004). The improved ability to colonize the rhizosphere enhances plant-bacteria interaction and increases the efficiency of beneficial mechanisms of PGPR (Altaf and Ahmad 2019; Guo et al. 2020; Santoyo et al. 2021).

In the presence of arginine in the growth media, MME3:SyNOS formed more biofilm than the control strain. This might possibly be due to the production of NO by the NOS_ox_ domain of SyNOS. Many studies have described NO as a signaling molecule in biofilm formation, acting both as an inducer or a dispersant of biofilms depending on the bacterial species. In *Pseudomonas aeruginosa*, which is considered a model for the study of biofilm formation in Gram-negative bacteria, it is well established that NO is able to disperse biofilms by lowering c-di-GMP levels (Cutruzzolà and Frankenberg-Dinkel 2016). In other species, such as *Pseudomonas simiae* (Vaishnav et al. 2016), the soil bacterium *Nitrosomonas europaea*, and the PGPR *Azospirillum baldaniorum* (Arruebarrena Di Palma et al. 2013), NO induces biofilm formation. Given that the role of NO in the development of fluorescent *Pseudomonas* biofilms has not been established yet, more research is needed to confirm if this molecule is behind the increase in biofilm formation of MME3:SyNOS. In addition, the fact that the difference in biofilm formation is evidenced when arginine is added is an interesting output as many amino acids have been identified in root exudates and arginine appears to play a key role in rhizosphere dynamics associated with recruited microorganisms (Oku et al. 2012). Also importantly, the *synos* expression did not negatively affect MME3 production of auxins or siderophores, both of them desirable properties in a PGPR.

To test the impact of *synos* expression on *P. koreensis* MME3-plant interaction, we selected the grass model *B. distachyon* as an experimental system for inoculation experiments. An analysis of* B. distachyon* root exudates proved the release of arginine to the rhizosphere and its participation in plant-bacteria chemotaxis (Saleh et al. 2020). Moreover, this plant shows phenotypic variation for some traits, including the responsiveness to different N availability (David et al. 2019).

Although inoculation with MME3:SyNOS improved vegetative growth of the aerial organs under LN-S, the effect produced was mostly on the root system. In semi-hydroponic growth, *synos* expression had a positive impact in root growth stimulation under both HN-Hy and LN-Hy. This positive effect on root growth was also observed in greenhouse experiment at 35 dpi under LN-S. These results evidenced a clear and constant overgrowth of roots inoculated with MME3:SyNOS bacterium. Under these evidences, we hypothesize that *B. distachyon* might be using the NO_3_^−^ provided by the bacterium through the function of the NOS_g_ domain of SyNOS to improve its growth. In agreement, David et al. (2019) demonstrated that greater availability of NO_3_^−^ from chemical fertilizer increases the dry weight of roots in *B. distachyon*.

At harvest, mean grain yields were higher in inoculated plants grown under HN-S and LN-S, but differences were not significant. The nitrogen accumulation in grain depends on both the N remobilization from leaf and the N uptake by root (Dupont and Altenbach 2003; Padhan et al. 2023). Our data highlight that the most important mechanism for N acquisition measured at the end of the culture cycle was soil N uptake, which in this study was boosted by MME3, regardless of the activity of SyNOS. Studies made in several genotypes of winter wheat (Bogard et al. 2010) and the results found by David et al. (2019) showed that grain N loading in *B. distachyon* cv. Bd21-3 is mainly based on post-anthesis N uptake, rather than N remobilization from photosynthetic sources.

In our case, N availability changed the N uptake, as this index had higher values under LN-S. Within this condition, the largest increase in N uptake occurred in inoculated plants. The available N varied in the inoculated condition, possibly because bacteria could influence the composition of the accompanying microbiota through processes of mineralization, denitrification, and volatilization, typical of the soil (Havlin et al. 2016). Coincident with our work, Kuang et al. (2022) showed that the inoculation of *B. distachyon* with *Herbaspirillum seropedicae* influenced the uptake of N from the medium by stimulating N depletion only in low N condition.

In a different approach, Del Castello et al. (2020) reported that transgenic *Arabidopsis thaliana* plants expressing the *synos* gene showed higher NUE values compared to control plants in both high- and low-N conditions. To analyze the potential agricultural impact of SyNOS enzyme in plant–PGPR association, we estimated the NUE of *B. distachyon* in response to inoculation with MME3:SyNOS in high- and low-nitrogen soils. The NUE of inoculated plants under LN-S was higher than that of non-inoculated plants grown in HN-S. This result agrees with Benedetto et al. (2017), who postulated that the improvement of N uptake depends on several factors: root size and morphology, root N transport system, and root interaction with microorganisms. Nitrogen utilization was improved by both *P. koreensis* MME3:c and MME3:SyNOS, possibly due to a greater soil exploring capability and greater adaptability to N deficiency in soils. The existence of a positive association between increased root weight and tissue N accumulation in our study cannot be ruled out. The higher NUE and NUpE found in inoculated plants suggest that MME3 could contribute to reduce N input by chemical fertilizer practices. Promoting a better use of N and reducing the application of fertilizers lead to reducing N losses and optimizing crop yields. This is a central goal for sustainable agriculture. We here demonstrated that the use of a PGPR with NOS activity to improve N cycling is a promising strategy to reach that goal. Still, more studies are needed to better understand the interaction of these rhizobacteria with plants and discover their true potential as inoculants.

## Data Availability

The datasets generated and/or analyzed during the current study are available from the corresponding author on reasonable request.
